# Antimicrobial Potential of Carvacrol against Uropathogenic *Escherichia coli* via Membrane Disruption, Depolarization, and Reactive Oxygen Species Generation

**DOI:** 10.3389/fmicb.2017.02421

**Published:** 2017-12-06

**Authors:** Imran Khan, Ashutosh Bahuguna, Pradeep Kumar, Vivek K. Bajpai, Sun C. Kang

**Affiliations:** ^1^Department of Biotechnology, Daegu University, Gyeongsan, South Korea; ^2^Department of Forestry, North Eastern Regional Institute of Science and Technology, Nirjuli, India; ^3^Department of Applied Microbiology and Biotechnology, Yeungnam University, Gyeongsan, South Korea

**Keywords:** urinary tract infection, carvacrol, *Escherichia coli*, extended-spectrum β-lactamase, reactive oxygen species, europathogen

## Abstract

Bacterial resistance to antibiotics poses a serious threat to cure diseases associated with microbial infection. Among the resistant bacteria, extended-spectrum β-lactamase (ESBL)-producing bacteria are the most concerned one as they encode the enzyme β-lactamase that confers resistance to most β-lactam antibiotics. The present study was carried out to determine the antimicrobial potential and the principle mechanism of action of carvacrol against ESBL *Escherichia coli* isolated from ascitic fluid of a patient having a urinary tract infection. Carvacrol exhibited a minimum inhibitory concentration (MIC) of 450 μg/ml at which it reduced *E. coli* cell counts significantly in a time-dependent manner. Carvacrol completely diminished the growth of *E. coli* after 2 h of incubation at its MIC. Fluorescent imaging displayed the elevated reactive oxygen species level and bacterial membrane depolarization leading to *E. coli* cell death in presence of carvacrol at its MIC. Furthermore, carvacrol displayed a severe detrimental effect on bacterial membrane disruption and cellular material release. In addition, a significant effect of carvacrol at sub-inhibitory concentration was observed on motility of *E. coli* cells and invasion of human colon HCT-116 cells in an *ex vivo* model. Based on the results, we conclude a potential antimicrobial role of carvacrol against ESBL *E. coli*.

## Introduction

Urinary tract infection (UTI), also known as bladder infection, commonly affects women compared to men due to several reasons such as female anatomy, sexual intercourse, family history, etc. Symptoms of UTI include pain in the lower abdomen, fever, fatigue, or bad smelling urine, etc. (Khawcharoenporn et al., 2013; Picozzi et al., 2014). It is important to recognize UTI at an early stage since it creates complications later on for treatment. The most common UTI microorganism is *Escherichia coli*, although others such as viruses and fungi have been reported (Khawcharoenporn et al., 2013). Multidrug-resistant (MDR) bacteria are common in UTI and make this type of infection more difficult to treat with routine antibiotics. In the recent years, extended-spectrum β-lactamase (ESBL) producing bacteria have become more common in UTI. These bacteria are resistant to the most β-lactam class of antibiotics due to the production of enzyme β-lactamase that selectively attacks the β-lactam ring of antibiotics (Khawcharoenporn et al., 2013; Picozzi et al., 2014). The β-lactamases are broadly classified into four classes A, B, C, and D on the basis of their amino acid sequences and named due to mutant forms of ESBL such as SHV, TEM, and CTXM- type β-lactamases. A variety of mutant lactamases have been discovered which reflect high resistance to β-lactam antibiotics (Bush and Jacoby, 2010).

The first ESBL microorganism was isolated in 1983 from Germany, after which a slow outbreak was reported worldwide. Many recent articles suggest that ESBL *E. coli* is one of the main causative agents of UTI (Khawcharoenporn et al., 2013). Ascites is an accumulation of abdominal fluid found in many elderly patients with UTIs and pathogenic bacteria are able to travel through the urine to ascitic fluid, which leads to infection. Therefore, it is important to find potential antimicrobial agents that are effective against ESBL *E. coli* (Abirami et al., 2012).

In recent years, there have been many reports on the use of essential oil or its components, as potent antimicrobials against a variety of pathogenic bacteria (Di Pasqua et al., 2007; Bajpai et al., 2009). Carvacrol, which is present in essential oils of *Origanum vulgare, Thymus vulgaris, Trachyspermum ammi, Lepidium africanum*, and *Citrus bergamia*, has been widely studied as an antimicrobial, antiviral, and anticancer agent (Johny et al., 2010; Dandlen et al., 2011; Nostro and Papalia, 2012; Monte et al., 2014; Honório et al., 2015). Carvacrol is a hydrophobic monoterpene in nature that easily penetrates the cell membranes of bacteria, leading to disruption of cell membrane integrity as well as release of bacterial cell contents (Cristani et al., 2007; Di Pasqua et al., 2007; Magi et al., 2015).

Therefore, the present study was carried out to evaluate the antimicrobial potential of carvacrol along with its role in the motility and invasion behavior of the ESBL *E. coli.*

## Materials and Methods

### Bacterial Strain and Chemicals

In the present study, ESBL-producing *E. coli* (KBN10P03217) was used which was isolated from the blood sample of the 70-years old male patient and exhibited resistance to several antibiotics, including ceftazidime, amphotericin B, ampicillin, ampicillin/sulbactam, aztreonam, cefazolin, cefepime, cefotaxime, ciprofloxacin, and trimethoprim/sulfamethoxazole. Bacterial sample was received after the ethical clearance from the ethical committee of Kyungpook National University Hospital, Daegu, South Korea. *E. coli* was grown in Luria-Bertani (LB) medium purchased from Acumedia (Lansing, MI, United States). Carvacrol, with a purity of ≥98%, was purchased from Sigma-Aldrich (St. Louis, MO, United States) and dissolved in 5% dimethyl sulfoxide (DMSO) (Sigma Aldrich). Phosphate-buffered saline (PBS) with 5% DMSO was used as a negative control for all experiments.

### Antibacterial Assay

The minimum inhibitory concentration (MIC) of carvacrol was evaluated using the broth micro-dilution technique in LB broth with an initial inoculum of 10^7^ cells/ml in a non-treated polystyrene microtiter plate CC7672-7596; (CytoOne) in accordance with the Clinical and Laboratory Standards Institute (CLSI). The MIC was interpreted as the lowest concentration of carvacrol that completely inhibited visible growth of the bacteria after 24 h of incubation at 37°C. Each concentration was tested in triplicate in at least three independent experiments.

### Time Kill Assay

The viable cell count was evaluated according to the method adopted by Chauhan and Kang (2014). Briefly, mid-log phase *E. coli* cells (10^7^ cells/ml) were inoculated into LB broth containing carvacrol (MIC) and incubated at 37°C. At different incubation times such as 0, 30, 60, 90, and 120 min, the culture was plated onto LB agar medium with appropriate dilutions, followed by incubation at 37°C for 24 h. Colonies were counted after the incubation period as indicated above. Simultaneously, the controls without carvacrol were also set up under the same experimental conditions.

### Fluorescence Microscopy Staining

Live and dead cells were evaluated by AO/EB (Acridine orange and ethidium bromide) fluorescent staining (Hameed et al., 2016). Overnight grown *E. coli* cells (10^7^ cells/ml) were treated with or without carvacrol (MIC) for 15 min at 37°C. After incubation, cells were harvested by centrifugation, washed with PBS and stained with AO/EB (1:1) (100 μg/ml) for 30 min. After 30 min incubation, cells were washed with PBS and visualized under fluorescent microscope. Similarly, to assess the effect of carvacrol on reactive oxygen species (ROS) and membrane potential, H_2_DCFDA [5(6) -Carboxy-2′,7′-dichlorofluorescein diacetate] and rhodamine 123 fluorescent staining was carried out, respectively (Warnes et al., 2012). In brief, *E. coli* cells (10^7^ cells/ml) were treated with or without carvacrol for 15 min followed by centrifugation to harvest the cells. After washing with PBS, cells were stained in dark for 30 min with H_2_DCFDA (10 mM) or rhodamine 123 (1 μg/ml). Cells were thoroughly washed with PBS and visualized under EPI fluorescent Eclipse TS100 Inverted Routine Microscope with PAXCam software (Nikon TS 100, Japan). All experiments were repeated three times independently and at least three different fields were observed for each culture.

### Crystal Violet Uptake Assay

Alteration of membrane permeability was detected by crystal violet assay (Devi et al., 2010). Suspensions of *E. coli* (10^7^ cells/ml) were prepared in LB broth. Cells were harvested at 10,000 rpm for 5 min. The cells were washed twice and suspended in 50 mM PBS (pH 7.4). Carvacrol at MIC was added to the cell suspension and incubated at 37°C for 30 min. Control samples were prepared similarly without treatment. The cells were harvested at 10,000 rpm for 5 min and suspended in PBS containing 10 μg/ml of crystal violet. The cell suspension was then incubated for 10 min at 37°C and centrifuged at 10,000 rpm for 5 min, after which the OD_590_ of the supernatant was measured using a UV-VIS spectrophotometer (UV-2120 Optizen, Mecasys, South Korea). The optical density (OD) value of crystal violet solution, which was originally used in the assay, was considered as 100% excluded. The percentage of crystal violet uptake for all samples was calculated using the following formula:

%Dye uptake = 100-[(OD of the sample/OD value of crystal violet solution) × 100].

### Cellular Material Release

Measurement of release of cellular materials (DNA) from *E. coli* cells was carried out by inoculating the mid-log phase culture (10^7^ cells/ml) into NaCl (0.85%) in the presence and absence of carvacrol (MIC) and incubated at 37°C. After incubation for 0, 30, 60, and 120 min, 1 ml of broth was transferred to an Eppendorf tube, which was centrifuged at 10,000 rpm. The absorbance of the supernatant was then measured at 260 nm using an UV-VIS spectrophotometer. Results were expressed in the form of OD recorded at each time interval. Simultaneously, the amount of released protein in the presence and absence of carvacrol was determined by using Bradford reagent (Devi et al., 2010). In brief, 5 ml overnight grown cells (10^7^ cells/ml) were harvested at 8,000 rpm, washed thoroughly three times using normal saline (0.85% NaCl) and finally suspended in 1 ml of saline in the presence of carvacrol. A control without carvacrol was also set up under the same experimental conditions. At different time points (60 and 120 min), protein release was quantified.

Furthermore, to confirm the damaging effect of carvacrol on *E. coli* cell membrane, the supernatant of carvacrol-treated bacterial suspensions was subjected to SDS-PAGE (Devi et al., 2010). Initially, bacterial cells were grown to OD 2.0 and then harvested at 10,000 rpm for 5 min at room temperature; the bacterial pellet was washed twice with PBS (pH 7.4) and re-suspended in PBS. Carvacrol at MIC value was added to the cell suspensions, and the samples were incubated at 37°C for 60 and 120 min. After treatment, the suspensions were centrifuged at 10,000 rpm for 10 min. Control samples were prepared similarly without carvacrol treatment. The supernatant (50 μl) was placed in a centrifuge tube, after which 25 μl of sample buffer (1 M Tris-HCl (pH-6.8), 50% glycerol, 10% SDS, 10% β-mercaptoethanol, 1% bromophenol blue) was added. The samples were then heated at 100°C for 5 min and loaded onto 12% SDS-PAGE. Silver staining was performed to analyze the proteins released due to membrane disruption.

### Scanning Electron Microscopic Examination

The potential effect of carvacrol on the cell morphology of *E. coli* was determined with the help of SEM (Khan and Kang, 2016). In brief, bacterial cells were incubated with or without carvacrol for 1 h, washed three times using PBS (pH 7.4), and then centrifuged at 4,000 rpm. The obtained pellet was suspended in PBS, and a thin smear was prepared on a glass slide, which was further fixed in glutaraldehyde (2.5 g/100 ml; Electron Microscopy Science, United States) for 2 h. After fixation, a dehydration step was performed using an increasing order of ethanol ranging from 50 to 100%, after which the cells were dried with liquid CO_2_. The dried cells were coated with gold in a sputter coater, and samples were observed under a scanning electron microscope (SEM) (Hitachi-S4300, Japan).

### Motility Assay

To assess the effect of carvacrol on bacterial motility, a colony grown on LB agar was stabbed into semi-solid medium (1% Bacto tryptone; Difco Laboratories, Detroit, MI, United States), 0.5% (w/v) NaCl, and 0.35% agar (Difco Laboratories, Detroit, MI, United States) with or without carvacrol at sub-inhibitory concentrations (100 and 150 μg/ml). Bacterial swarming was assessed after incubating the stabbed *E. coli* at 37°C for 24 h. Motility was further confirmed by the non-quantitative hanging-drop technique (Inamuco et al., 2012). In brief, a droplet of bacterial culture was suspended from a glass coverslip over a microscope slide with a central concavity. The motility of bacterial cells was observed at a magnification of 100x under a light microscope.

### Growth Conditions and Cell Lines

Human colon HCT-116 cell line was purchased from the Korean Cell Line Bank (KCLB) and cultured in RPMI-1640 medium at 37°C in 5% CO_2_ incubator.

### Invasion Protection Assay

*Escherichia coli* was grown to mid-log phase in the presence of 100 or 150 μg/ml of carvacrol. Bacterial culture was centrifuged and suspended in RPMI-1640 medium to a density of 1 × 10^8^ cells/ml. HCT-116 colon cells were grown to confluence in 12-well tissue-culture plates, washed three times with plain medium, and incubated with 1 ml of the bacterial solution for 1 h at 37°C. HCT-116 monolayers in wells were washed once with 1 ml of warm plain medium and then incubated for 2 h with 1 ml of 100 mg/ml of gentamycin in warm RPMI-1640 medium to kill extracellular bacteria. Cells were washed three times with plain RPMI-1640 medium and finally lysed with 1% Triton X-100. The number of intracellular bacteria was determined by colony-plating as described above (Khan and Kang, 2016).

### MTT Assay

The cytotoxic effect of carvacrol was first determined by MTT assay (Khan and Kang, 2017) Briefly, 5 × 10^4^ HCT-116 cells/well were incubated in the presence of carvacrol at the concentrations of 50, 100, and 150 μg/ml for 2 h at 37°C in a CO_2_ incubator. After incubation, cells were treated with MTT solution (5 mg/ml) to produce dark blue colored formazan crystals, which were further dissolved in 50 μl of DMSO. Finally, absorbance was measured at 540 nm in a microplate reader (Bio-Tek Instrument, Co., Everett, WA, United States).

### Statistical Analysis

All experiments were carried out in triplicate. The results were expressed as mean ± standard deviation (SD) of three independent experiments. Statistical significance was calculated using Student’s “*t”* tests. ^∗^Represents *P*- values < 0.05, ^∗∗^represents *P*- values < 0.01, and ^∗∗∗^represents *P*-values < 0.001.

## Results

### Time Killing Assay

**FIGURE 1:**
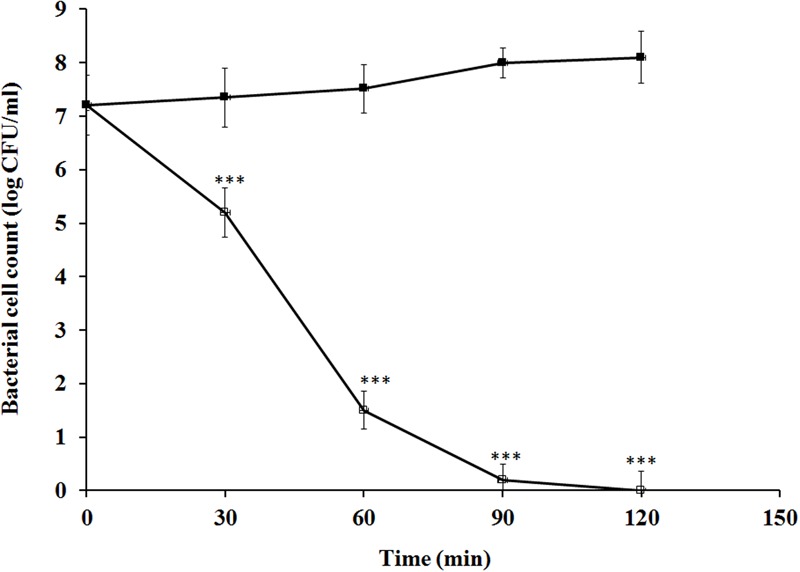
Effects of carvacrol (450 μg/ml) on viable cell counts of *Escherichia coli* at various time intervals. The data are represented as the means ± SD of three independent experiments, ^∗∗∗^*P* < 0.001 vs. control.

In this assay, carvacrol exhibited a severe detrimental effect on the growth of *E. coli* at MIC (450 μg/ml). Especially, viable cell counts of *E. coli* decreased significantly in the time-dependent manner after exposure of carvacrol. As depicted in **Figure 1**, log CFU/ml drastically decreased to less than log 2 CFU/ml after 60 min, followed by complete inhibition at 120 min. This proves that carvacrol has a severe toxic effect on the viable count of *E. coli*, whereas cell number of control sets prepared without carvacrol increased slightly.

### Live/Dead Bacterial Cells Evaluation

**FIGURE 2:**
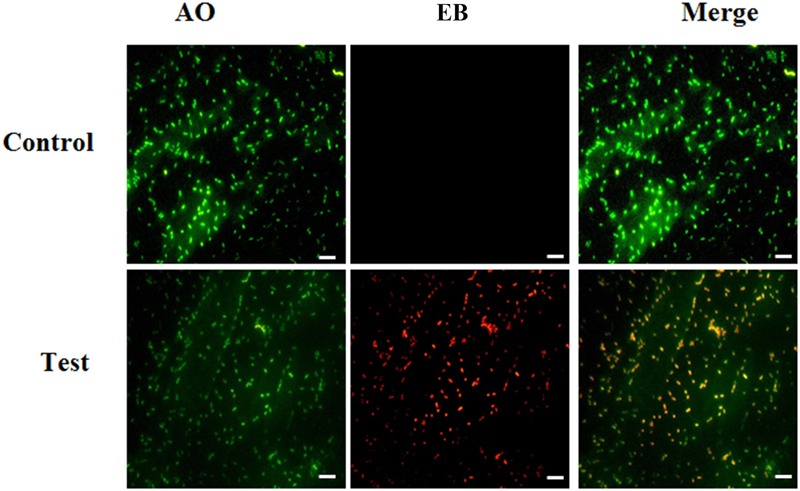
Microscopic assessment of *E. coli* after treatment with carvacrol. The bacterial cell death was examined *via* AO/EB staining and fluorescent microscopy. Live bacteria emit green fluorescence, and dead bacteria as red fluorescence [Scale bar = 10 μm].

Furthermore, live and dead bacterial cells were evaluated by AO/EB staining. In AO/EB staining, AO stains live cells, however; EB stains to cells which have lost membrane integrity. AO stained cells give green fluorescence representing live cells while EB gives red fluorescence denoting dead cells. We found a high red color fluorescence in carvacrol treated cells, indicating its toxicity to *E. coli* (**Figure 2**).

### Reduction of Membrane Potential and ROS Generation in *E. coli* by Carvacrol

**FIGURE 3:**
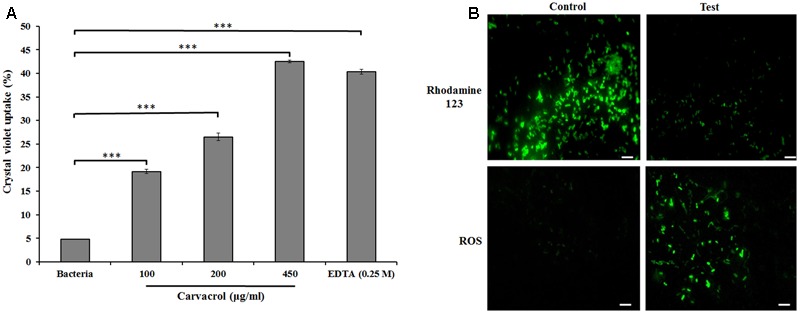
**(A)** Crystal violet uptake of carvacrol (100–450 μg/ml)-treated *E. coli.* EDTA (0.25 M), was used as a positive control. The data are represented as the means ± SD of three independent experiments, ^∗∗∗^*P* < 0.001 vs. control. **(B)** Effect of carvacrol on *E. coli* membrane potential, using lipidophilic stain rhodamine 123 (Rh123). Bacterial cells were incubated with or without carvacrol (450 μg/ml) for 15 min and then stained with Rh123. Cells with intact membranes stain brightly due to accumulation of Rh123, however if the membrane is compromised the potential is lost and staining dissipates. Generation of reactive oxygen species (ROS) in *E. coli* by H_2_DCFDA staining. Bacterial cells were incubated with or without carvacrol (450 μg/ml) for 15 min then stained with H_2_DFFDA [Scale bar = 10 μm]. All the fluorescent images visualized under EPI fluorescent Eclipse TS100 Inverted Routine Microscope with a PAXCam software (Nikon TS 100, Japan).

A crystal violet uptake assay was used to determine the membrane permeability. Uptake of crystal violet by *E. coli* was 4.8% in the absence of carvacrol which increased by 26 and 42% at 200 and 450 μg/ml (MIC) of carvacrol treatment, respectively (**Figure 3A**). Furthermore, we investigated the membrane polarization with rhodamine 123. Carvacrol treatment to *E. coli* for 15 min significantly reduced the fluorescence intensity of rhodamine 123 (**Figure 3B**) signifying its adverse effect on electrical potential (ΔΨ) of bacterial membrane. Moreover, the role of carvacrol on the induction of oxidative stress by the involvement of ROS was observed by H_2_DCFDA staining (**Figure 3B**). The fluorescence dye H_2_DCFDA was used to detect the accumulated ROS in the bacterial cell where it is deacetylated by esterase and gives green color that visualized under fluorescence microscope. After treatment with carvacrol for 15 min, ROS level found to be elevated in the *E. coli* representing its impact on the induction of oxidative stress.

### Effect of Carvacrol on Cellular Material Release from *E. coli*

**FIGURE 4:**
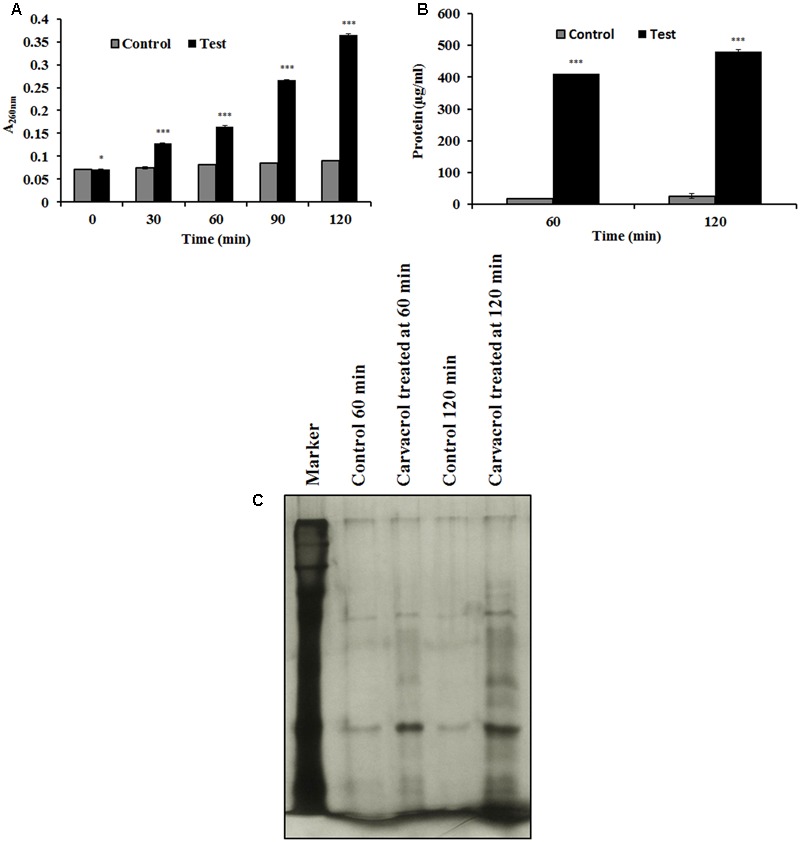
**(A)** Effects of carvacrol on release of cellular materials from *E. coli* based on absorbance value recorded at 260 nm. **(B)** Protein release due to carvacrol treatment. **(C)** Time-dependent proteins released into supernatant by carvacrol treatment (MIC), detected by using SDS-PAGE. Lane-2 and lane-3 represent control and carvacrol treatment at 60 min; lane-4 and lane-5 represent control and carvacrol treatment at 120 min, respectively. Bar diagrams are depicted as the means ± SD of three independent experiments, ^∗^*P* < 0.05, ^∗∗∗^*P* < 0.001 vs. control.

In this assay, carvacrol showed potential to disrupt the cell membrane integrity of *E. coli*, leading to release of cellular materials. As depicted in **Figure 4A**, carvacrol led to the release of intracellular materials based on absorbance at 260 nm, whereas control experimental sets showed no release of cellular contents. Simultaneously, protein release was detected by using the Bradford method. Protein release serially increased at 60–120 min from 400 to 480 μg/ml, in the carvacrol treated cells (**Figure 4B**). To confirm membrane disintegration of *E. coli* by carvacrol, released membrane proteins were also detected by SDS-PAGE. Intensities of separated bands increased from 60 to 120 min and showed a distinct banding pattern as compared to control (untreated) (**Figure 4C**).

### Scanning Electron Microscopy

**FIGURE 5:**
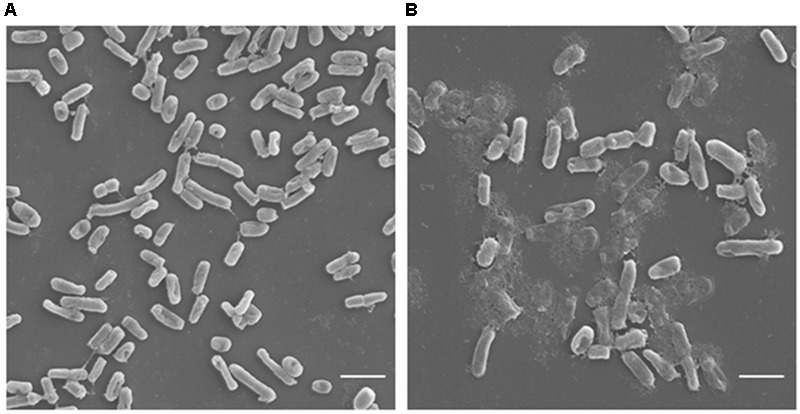
Scanning electron microscopic analysis of *E. coli.* **(A)** Represents cells without any treatment showing regular and intact cell morphologies. **(B)** Cells treated with carvacrol at MIC (450 μg/ml) showing distorted bacterial cell wall morphology (Scale bar = 2.5 μm).

Scanning electron microscopy was carried out to visualize the effect of carvacrol on cell morphology of *E. coli*. Carvacrol caused cell membrane lysis, as evident by membrane damage and altered cell morphology unlike to control (untreated) where intact cells were observed (**Figure 5**).

### Reduction of Motility and Colon Cell Protection by Carvacrol

**FIGURE 6:**
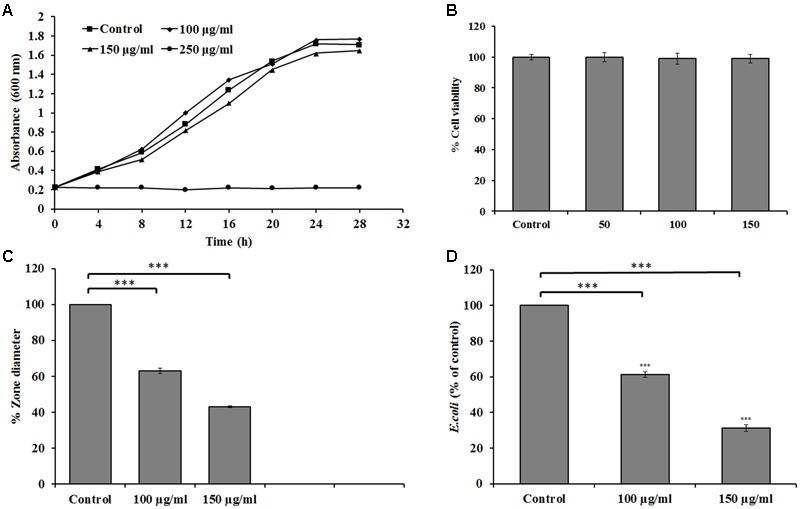
**(A)** Swarming of *E. coli* in soft agar plates. **(A)** Representative growth curve of *E. coli* in the presence of different concentrations of carvacrol [0 (control), 100, 150, and 250 μg/ml]. Each reading measured in triplicates and in three independent experiments. **(B)** MTT assay evaluation of carvacrol on HCT-116 colon cells after 2 h. **(C)** Relative zone diameter compared to the control without carvacrol. **(D)** Invasion of colon cells by *E. coli*, presented as % relative to control without carvacrol (100 and 150 μg/ml). The data are represented as the means ± SD of three independent experiments, ^∗∗∗^*P* < 0.001 vs. control.

To determine the suitable concentration of carvacrol for motility and invasion experiments, we plotted a growth curve with different concentrations of carvacrol (0–250 μg/ml). As a results, no significant growth inhibition of *E. coli* was observed at 100 and 150 μg/ml (**Figure 6A**). Moreover, carvacrol did not exert the cytotoxic effect on HCT-116 colon cells as determined by MTT assay (**Figure 6B**). Furthermore, carvacrol showed a significant effect on the bacterial motility at the sub-lethal concentrations of 100 and 150 μg/ml. At 150 μg/ml concentration of carvacrol, *E. coli* showed minimum motility with 39% relative zone diameter as compared to 100% in the control (untreated) (**Figure 6C**). Light microscopy results of the hanging drop assay revealed complete loss of motility at concentration 150 μg/ml of carvacrol (data not shown). Furthermore, *E. coli* cells pretreated with carvacrol showed decreased invasion to HCT-116 colon cells as compared to control (untreated) (**Figure 6D**). *E. coli* cells pretreated with 150 μg/ml carvacrol represent 31 ± 3.2% invasions as compared to the 100% invasion of the control.

Two concentrations of carvacrol 100 and 150 μg/ml were considered as sub-lethal as bacterial cells were found to grow freely in these concentrations. The finding suggests that carvacrol has strong inhibitory effects against bacterial invasion and motility.

## Discussion

Urinary tract infection is one of the most common diseases worldwide and occurs mainly in women. In UTIs, the microflora is mainly composed of *E. coli, Klebsiella pneumoniae*, and *Pseudomonas aeruginosa* and is predominated by *E. coli* (Khawcharoenporn et al., 2013; Picozzi et al., 2014). These bacteria are able to cause issues due to anatomical changes and poor maintenance of personal hygiene during pregnancy. Moreover, *E. coli* has a unique structure that allows it to attach to uroepithelial cells, invade, and spread intracellularly, ultimately leading to pyelonephritis (Khawcharoenporn et al., 2013). Furthermore, *E. coli* has the ability to travel way through the urinary tract to ascites, resulting in ascitic fluid infection (Abirami et al., 2012). Due to the severity of *E. coli* infection and its resistance toward β-lactam antibiotics, a potent agent with the ability to kill this uropathogen and simultaneously protect mammalian cells from infection is needed.

Natural compounds are used to treat infectious diseases worldwide (Ndjonka et al., 2013). Most of these compounds are hydrophobic, which grants strong antimicrobial properties against Gram-negative bacteria. Many essential oils and their components have antimicrobial activities against foodborne pathogens, with carvacrol used in the food industry as a food additive (Cristani et al., 2007; Yu et al., 2012; Yang et al., 2015). In our study, we used carvacrol due to its hydrophobic nature and its potential as an antimicrobial agent (Nostro and Papalia, 2012). Our results suggest that carvacrol has potent antimicrobial activity against ESBL E. coli isolated from ascetic fluid of UTI patients, as it reduced the viable bacterial count at MIC. Previously, similar kinds of inhibitory effects of carvacrol have been reported against *Salmonella enteritidis* and *Campylobacter jejuni* (Johny et al., 2010).

Any effective antimicrobial compound must either penetrate or disrupt the bacterial plasma membrane. In the present study, carvacrol disrupted the *E. coli* membrane, leading to release of cellular contents as confirmed based on the absorbance values measured at 260 nm. Most of antimicrobial agents rupture bacterial membrane *via* membrane disintegration and generation of ROS. Here, we observed that carvacrol induces enhanced crystal violet uptake which denotes that there was an increasing level of membrane leakage, thus changed the membrane permeability. Moreover, the membrane depolarization was also validated using rhodamine 123 fluorescence dye. During the initiation of cell death mechanism, ROS generation found to play a crucial role to initiate cessation of cell. In this study, we confirmed the enhanced level of ROS in *E. coli* cells after treatment with carvacrol using H_2_DCFDA dye. The finding suggests a highly oxidative stress environment inside the *E. coli* in response to carvacrol. A high oxidative stress is well-associated with adverse effect on cell components such as DNA and protein (Warnes et al., 2012).

Furthermore, SEM analysis revealed that carvacrol interacted with the lipid bilayer of bacterial cells and induced structural disruption. These results are similar to the previous findings where thymol as an isomer of carvacrol was able to disrupt the bacterial plasma membrane and caused the release of cellular contents (Chauhan and Kang, 2014).

Although some antibiotics are effective in treating UTI-causing pathogens, but fail to provide protection against bacterial virulence such as invasion or intracellular killing. Therefore, an antimicrobial should be able to inhibit the motility of bacteria in order to protect mammalian cells from invasion. Hence, we evaluated the role of carvacrol to protect colon cells from *E. coli* invasion. To achieve this, we treated *E. coli* cells with a sub-lethal concentration of carvacrol and carried out infection of colon cells. Carvacrol effectively reduced motility and invasion, which showed a positive correlation. The data obtained from this study indicate that carvacrol has the ability to protect colon cells from *E. coli* invasion and could affect the functionality of the bacterial flagellum, which is in accordance with a study done by Inamuco et al. (2012).

## Conclusion

Carvacrol has the ability to disrupt the membrane integrity of *E. coli*, resulting in the release of cellular contents (DNA and proteins), and protect colon cells from bacterial invasion. Based on findings, it can be concluded that carvacrol could be a potent antimicrobial candidate against ESBL *E. coli* causing UTIs in women population. Though there have been many previous reports on the antimicrobial action of various agents against *E. coli*, this is the first report on the action of carvacrol against UTI-causing ESBL *E. coli*, and its effects on motility and invasion.

## Author Contributions

IK and AB: Designed and performed the experiments and initially wrote the manuscript. PK, VB, and SK: Critically edited and finalized the manuscript.

## Conflict of Interest Statement

The authors declare that the research was conducted in the absence of any commercial or financial relationships that could be construed as a potential conflict of interest.
